# A case of silent invasion: Citizen science confirms the presence of *Harmonia axyridis* (Coleoptera, Coccinellidae) in Central America

**DOI:** 10.1371/journal.pone.0220082

**Published:** 2019-07-18

**Authors:** Thomas Hiller, Danny Haelewaters

**Affiliations:** 1 Institute of Evolutionary Ecology and Conservation Genomics, University of Ulm, Ulm, Germany; 2 Department of Organismic and Evolutionary Biology, Harvard University, Cambridge, Massachusetts, United States of America; 3 Department of Zoology, University of South Bohemia, České Budějovice, Czech Republic; University of Manitoba, CANADA

## Abstract

*Harmonia axyridis* (Coleoptera, Coccinellidae) is a globally invasive ladybird. It has been intentionally introduced in many countries as a biological control agent, whereas it has been unintentionally released in many others. Climatic factors are important in limiting the spread of *H*. *axyridis*. For example, very few records are known from tropical or desert regions. Currently, no published reports are known from Central America. Here, we report *H*. *axyridis* from Costa Rica, Guatemala, Honduras, Panama, and Puerto Rico. Specimens were either observed by the authors, discovered in dried insect collections, or retrieved from searching through online photographs available from the citizen science project iNaturalist and the photo-sharing website Flickr. These new records and the wide distribution of *H*. *axyridis* in Latin America suggest several invasion events, which have gone unnoticed until now. We stress the need for further, large-scale monitoring and show the advantage of citizen science to assess the presence of invasive alien species.

## Introduction

Citizen scientists, non-professionals who engage in scientific investigations, are of all ages. The field of citizen science has been gaining more traction in recent years and is becoming more popular and respected among ecologists and environmental scientists [1,2]. In fact, scientific research before the end of the 19th century was mostly conducted by amateurs [3], often experts in their area of work. Especially during the last 150 years amateur scientists have become increasingly marginalized, whereas the sciences professionalized [4]. However, there are examples in which citizen science shows incredible value. Projects that focus on large-scale ecological questions, often rely on citizen science input to offer simultaneous coverage of large geographic areas for the generation of useful datasets. Such projects might otherwise not be manageable by professional scientists alone due to logistical reasons and also financial and time constraints [4]. Examples are the North American Breeding Bird Survey (https://www.pwrc.usgs.gov/bbs/), the UK Ladybird Survey (http://www.ladybird-survey.org/) [5,6], and the Reed Life Survey (http://reeflifesurvey.com).

Recent technical developments are increasingly providing unpredicted possibilities for citizen science initiatives. Mobile devices come by default with high-resolution cameras and built-in GPS sensors, and combined with applications such as iNaturalist (http://www.inaturalist.org), they allow the user to easily connect and submit high-quality observations. However, many applications, e.g., eBird [7,8], require a certain level of previous expertise to participate and submit data, aiming at more experienced amateur scientists. Getting started can therefore sometimes be tricky for unexperienced hobby naturalists. If no previous training is included for volunteers, especially easy recognizable taxa are ideal for citizen science projects aiming at a broader field of participants. Other platforms, like iNaturalist, depend on community identifications of submitted contributions, encouraging users, regardless of their level (amateur or professional), not only to interact with each other, but also to function as a quality filter of the resulting dataset.

The combination of (often) easy recognition and scientific urge for knowledge have made the topic of invasive alien species a flagship for many citizen science projects, logging the occurrence and distribution through time. For example, the European Alien Species Information Network (EASIN) launched a smartphone application introducing 48 invasive species of concern and allowing to report sightings, view sightings maps, and review personal species records [9]. Not included in EASIN, but unquestionable of ecological importance to the native environment is the globally invasive harlequin ladybird, *Harmonia axyridis* (Coleoptera, Coccinellidae) [6,10].

Being a predatory insect, *H*. *axyridis* plays a principal role in natural pest control regulating the population density of insect pests. However, when introduced into new ecosystems it can induce unanticipated and undesirable effects [10,11]. This ladybird, native to eastern Asia [10], has been intentionally introduced, often repeatedly, in several areas of Europe, North and South America, and Africa as a biological control agent. Nowadays it is established in many countries outside of its native range, most recently also in New Zealand [10,12]. Note that wild populations of *H*. *axyidis* in South America and Africa are the result of unintentional release, (most likely) from a single eastern North American bridgehead population [13]. *Harmonia axyridis* competes with native predators and parasitoids for common food resources and is efficient in intraguild predation. It has become a concern and a threat, because with increasing density of *H*. *axyridis* populations, native diversity is under pressure [6,14,15,16]. In addition, *H*. *axyridis* also has serious impacts in the food processing industry, particularly in wine production; just a few individuals hidden between the grapes are enough to contaminate the flavor of wine through their reflex bleeding [11,17].

*Harmonia axyridis* naturally occurs in temperate and subtropical regions [15,18] and the distributional pattern of its invasive populations in Europe suggest it should not survive or develop at high temperatures. Knapp and Nedvěd [19] found that extended exposure to 33°C significantly decreases hatching and survival rates of several developmental stages of Central European specimens of *H*. *axyridis*. Similarly, Benelli and colleagues [20] found that fecundity and fertility of Italian *H*. *axyridis* were decreased at 30°C compared to 25°C. There are several reports of *H*. *axyridis* from tropical South America, such as in Colombia, Ecuador, and Peru [21] probably thanks to the mild climate at high elevations. In tropical lowlands, records are relatively scarce: Brazil [22] in South America, and Kenya [18] and Tanzania [23] in Africa. In these countries, establishment to viable populations in the wild has been suggested to be unlikely because of susceptibility to high temperatures [19]. The same is true for records in hot desert climates, which is the case in Saudi Arabia where a specimen was collected in 2005 [24]. Another presumed reason for failure to establish in southern latitudes is the scarcity of prey [25,26].

In this study we report *H*. *axyridis* from tropical Central America based on personal observations and dried specimens from museum collections. We mapped its current distribution in Central and South America, including the Caribbean, by gathering records from iNaturalist and the photo-sharing website Flickr.

## Materials and methods

### Collections by the authors

**Fig 1:**
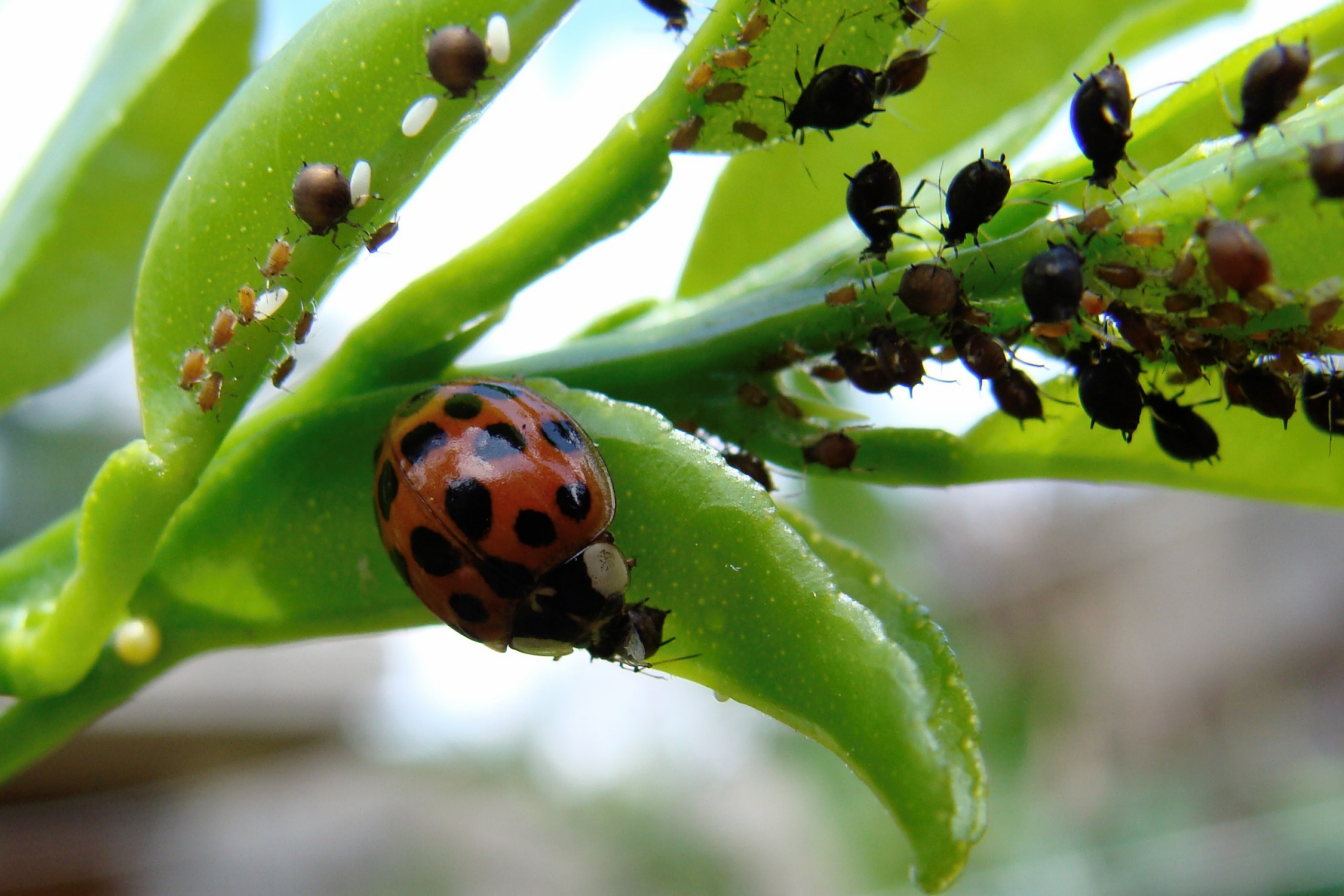
A specimen of *Harmonia axyridis* observed in Costa Rica.

With permission of the landowners, around 15 individuals of *H*. *axyridis* were observed in September 2009 on the campus of the University of Costa Rica (UCR: 9.937201, -84.050332) as well as in a close-by private garden (9.935214, -84.059777) on an aphid-infested citrus tree (Fig 1). No animal was handled during this study, only passively observed and therefore no research permit from local authorities was required. Pictures of one individual were uploaded to iNaturalist to create an accessible public record. We also searched through dried insect collections for specimens of *H*. *axyridis* that had gone unnoticed thus far. We screened insect collections at the Department of Biology at UCR, the Museo Nacional de Costa Rica (MNCR), and at Tupper Center at the Smithsonian Tropical Research Institute in Panama (STRI).

### SCAN data portal

We searched through the online Symbiota Collections of Arthropods Network (SCAN) repository of occurrence data for arthropods (http://scan-bugs.org/portal/) in North American collections. Searching for “Coccinellidae” and “Panama” resulted in 254 records, of which 218 were not identified to genus level. We reached out to the curators of the collections at which these unidentified ladybirds were deposited: Essig Museum of Entomology, University of California, Berkeley (1 specimen); Stuart M. Fullerton Collection of Arthropods, University of Central Florida (180 specimens); Entomology collection at University of Kansas Biodiversity Institute & Natural History Museum (6 specimens); and C.A. Triplehorn Insect Collection, Museum of Biological Diversity, Ohio State University (31 specimens). We asked curators to check whether the unidentified specimens in their collection were *H*. *axyridis*.

### Online data collections

We widened our search for Latin American records to iNaturalist (http://www.inaturalist.org) and Flickr (https://www.flickr.com/). Information from iNaturalist was extracted with the help of the R package *rinat* [27], by using the function get_inat_obs() and the search parameter “*Harmonia axyridis*”. The resulting list of observations was inspected for correct species identification. Additionally, we manually screened unidentified Coccinellidae of the entire geographical region submitted to iNaturalist and included records not discovered by the automatic search inquiry. We conducted manual photo searches on Flickr, an image-hosting site through which users can showcase and comment on submitted pictures. The search queries used were “Asian Lady Beetle, “Asian Ladybird”, “Harlequin Lady Beetle”, “Harlequin Ladybird”, “Multicolored Lady Beetle”, “Multicolored Ladybird”, “*Harmonia axyridis*”, “Coccinellidae”, “mariquita”, “catarina”, and “joaninha”. The results were again inspected for correct species identification. When displaying *Harmonia axyridis*, ID of the picture, location, date of the observation, and username were extracted. All manual searches were conducted in July 2018, whereas the automated search for *Harmonia axyridis* was updated last on 15 December 2018. Furthermore, we created an automatically updating project on iNaturalist (https://www.inaturalist.org/projects/harmonia-axyridis-in-latin-america/), allowing to monitor newly available records and the spread of *H*. *axyridis* in Latin America. Observations are only added to the project when they are “research grade.” This means that they have GPS coordinates and that the iNaturalist community agrees with the identification made by the person who created the record.

All observations were illustrated using ggplot() implemented in the R package *ggplot2* [28], showing the current distribution of *H*. *axyridis* in Latin America.

## Results

**Fig 2:**
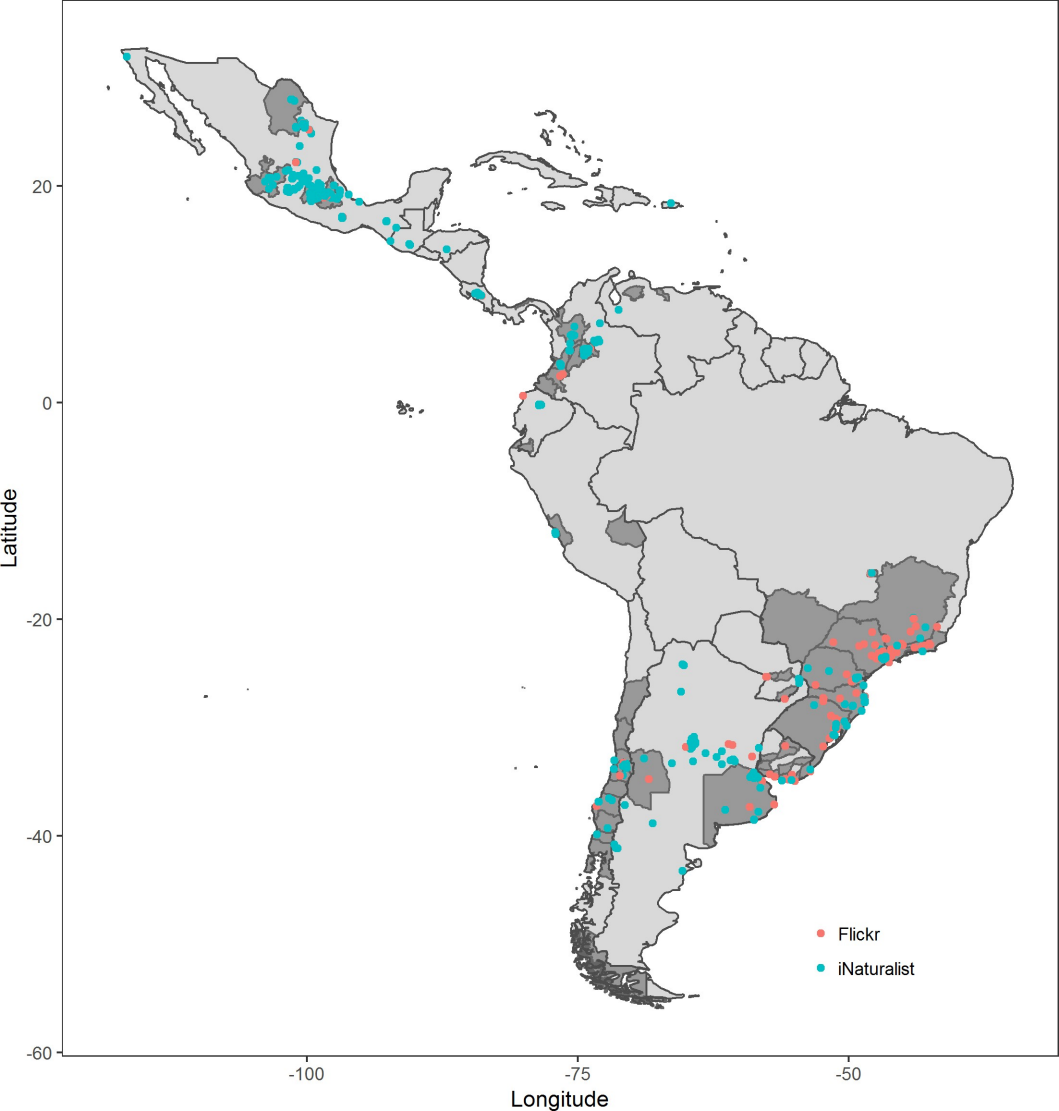
Occurrence of *H*. *axyridis* in Latin America. Red dots mark observations obtained from Flickr, blue dots mark observations obtained from iNaturalist, while the known distribution based on records in the literature is represented by dark-gray shadings.

We report here a total of 1096 individual records of *H*. *axyridis* and show a wide distribution of this species in Central and South America (Fig 2, S1 Dataset). The revision of museum specimens led to a total of 30 individual records of *H*. *axyridis* for Costa Rica, dating as far back as 1988 (Table 1). Our search inquiry on iNaturalist resulted in a total of 856 records from 14 countries: Argentina (89 records), Brazil (121), Chile (40), Colombia (123), Costa Rica (14), Ecuador (12), Guatemala (2), Honduras (1), Mexico (445), Paraguay (1), Peru (3), Puerto Rico (1), Uruguay (3), and Venezuela (1). On Flickr we found a total of 210 records from 9 countries: Argentina (24), Brazil (116), Chile (11), Colombia (12), Costa Rica (3), Ecuador (3), Mexico (23), Paraguay (2), and Uruguay (16). Of the 218 unidentified Panamanian ladybirds from insect collections revealed through SCAN, none were *H*. *axyridis* (Zachary H. Falin, Louis S. Hesler, Sandor Kelly, Peter T. Oboyski, Barbara J. Sharanowski, pers. comm.).

**Table 1:** Earliest records of *H*. *axyridis* in Latin America, its occurrence based on online available data and museum collections, and first mentions in the literature.

COUNTRY					
Department or Province	Year(s) earliest record	iNaturalist	Flickr	Museum	Literature
ARGENTINA					
Buenos Aires	2001	2006, 2010–2011, 2015–2018	2006–2008, 2010–2011, 2013–2014, 2016		[29,30]
Chubut	2017	2017			
Ciudad de Buenos Aires	2007	2017–2018	2007, 2011, 2014		
Córdoba	2007	2015–2018	2007		
Entre Ríos	2007	2015	2007		
Jujuy	2016	2016, 2018			
Mendoza	2011	2018	2011		[30]
Misiones	2016	2018	2016		
Neuquén	2014	2014, 2018			
Río Negro	2014	2014, 2018			
San Luis	2018	2018			
Santa Fe	2008	2010, 2012–2014, 2017–2018	2008–2009, 2014		
Tucumán	2018	2018			
BRAZIL					
Distrito Federal do Brasil	2009	2018	2009–2011		[31,32]
Espirito Santo	2011		2011		
Mato Grosso do Sul	2010				[33]
Minas Gerais	2006	2017–2018	2008, 2010–2012		[34]
Paraná	2002	2012, 2017–2018	2006–2007, 2009–2012		[22,35,31]
Rio de Janeiro	2006	2012, 2017–2018	2007, 2010–2011, 2013–2014, 2016		[36]
Rio Grande do Sul	2006	2016–2018	2006–2015		[33]
Santa Catarina	2011	2012, 2018	2011–2012		[33]
São Paulo	2004	2010–2011, 2015–2018	2005, 2007–2014, 2016–2018		[37]
CHILE					
Atacama	2014				[15]
Auracanía	2011	2017–2018			[15]
Bío Bío	2013	2018	2015		[15]
Coquimbo	2009				[15]
Magallanes	2015				[15]
Maule	2012				[15]
Metropolitana de Santiago	2008	2010, 2013, 2017–2018	2010–2012, 2014		[30]
Valparaíso	2008	2016, 2018			[30]
Libertador General Bernardo O'Higgins	2010	2018	2014		[15]
Los Lagos	2014/2015				[15]
Los Ríos	2013	2018			[15]
COLOMBIA					
Antioquia	1998	2016–2018	2010–2012, 2014, 2016		[21]
Boyacá	2016	2016–2018			
Caldas	2005	2017			[21]
Cauca	1994		2010		[21]
Cundinamarca	2001	2015–2018	2013–2015		[21,38]
Nariño	1989				[21]
Risaralda	2017	2017–2018			
Santander	2018	2018			
Tolima	2005				[21]
Valle del Cauca	1999	2014, 2017–2018			[21,39]
COSTA RICA					
Alajuela	1996	2017–2018		1996, 2001, 2012	
Cartago	2015	2017–2018	2015		
Heredia	1996	2014	2009	1996, 2001, 2004, 2007, 2011	
San José	1988	2009, 2011, 2017–2018	2011	1988, 1997, 1999, 2004–2005, 2007, 2015	
ECUADOR					
Esmeraldas	2015		2015		
Loja	2012				[40]
Pichincha	2011	2018	2011, 2017		
GUATEMALA					
Guatemala	2017	2017–2018			
HONDURAS					
Francisco Morazán	2018	2018			
MEXICO					
Baja California	2017	2017			
Chiapas	2015	2015–2018			
Coahuila	2006^a^	2010–2011, 2016, 2018			[11]
Distrito Federal	2002^a^	2010, 2013–2018	2006–2008, 2011, 2013		[41]
Guanajuato	2014	2014–2018			
Hidalgo	2014	2014, 2018			
Jalisco	2006^a^	2013–2018			[11]
Mexico	2006^a^	2014–2018	2007		[11]
Michoacán	2012	2012, 2014, 2016–2018			
Morelos	2000^a^, 2006^a^	2014–2018	2014		[11,42^b^]
Nuevo León	2006	2013–2018	2006–2007, 2011–2012, 2014–2015		
Oaxaca	2014	2014–2015, 2018	2015		
Puebla	2006^a^	2010–2012, 2017–2018	2011, 2013		[11]
Querétaro	2010	2011, 2014–2018	2010, 2015		
San Luis Potosí	2012	2015–2017	2012		
Tlaxcala	2015	2015–2018			
Veracruz	2013	2013–2014, 2016–2018			
PANAMA					
Colón	2014^a^				[43]
PARAGUAY					
Alto Paraná	2017	2017			
Asunción	2010		2010		
Caaguazú	2006				[44]
Central	2010		2010		
Cordillera	2007				[44]
Itapúa	2006				[44]
PERU					
Callao	2011				[45]
Lima	2010^a^	2016, 2018			[30,45]
Madre de Dios	2011				[45]
Tumbes	2010^a^				[30,45]
PUERTO RICO					
Puerto Rico	2017	2017			
URUGUAY					
Canelones	2007		2014–2015, 2017		[46,47]
Colonia	2008		2008, 2010		[47]
Florida	2006				[47]
Lavalleja	2010		2010		
Maldonado	2009	2018	2009		[47]
Montevideo	2009	2018	2009		[46]
Río Negro	2011				[47]
Rivera	2012				[47]
Rocha	2010	2018	2010		[47]
San José	2012		2015		[47]
Soriano	2012				[47]
Tacuarembó	2011		2011		[47]
Treinta y Tres	2011				[47]
VENEZUELA					
Aragua	2014^a^				[48]
Lara	2014^a^				[48]
Mérida	2017	2017			

^a^ Year of publication is used for records missing to report date of observation.

^b^ Source retrieved from [43].

Concerning the Caribbean Islands, we obtained a single record from Puerto Rico (iNaturalist, https://www.inaturalist.org/observations/9637737). On the Latin American mainland, *H*. *axyridis* has now been reported in all countries except for Belize, El Salvador, Nicaragua (Central America), Bolivia, French Guiana, Guyana, and Suriname (South America). The presence of *H*. *axyridis* in Panama is here revealed but was already published in a regional journal in Spanish language [43].

## Discussion

### Alternative data sources for biological records

Both natural history collections and citizen science projects are alternatives for systematic biological surveys of given species. Natural history collections harbor billions of specimens of which many are associated with taxonomic, geographic, and temporal data. These collections are an important asset in the study of the world’s past and current biodiversity, to understand changing parasite–host dynamics, reconstruct evolutionary history of infectious agents, provide data on phenological changes of organisms in response to climate change, identify unknown specimens and discover undescribed species, determine when pests, pathogens, or vectors are introduced, etcetera [49,50,51,52,53]. All too often, natural history collections are only accessible by researchers of the institutions where they are housed [54], resulting in significantly understudied collections [55] and an estimated average “shelf life”–the time between discovery and description of a new species–of 21 years [56]. Only an estimated 3% is digitized of the 1.2–2.1 × 10^9^ specimens, lots, and collections [57]. As a result, most of the natural history collections around the world are not virtually accessible. Digitation, on the other hand, is linked to an immense effort in both financial and labor-intensive terms [58]. Using traditional methodology, digitation of all natural history collections has been estimated at €150,000 million (~ $170,000 million) and 1,500 years [55]. Therefore, using new technologies and modern workflows as well as collaborative, web-based collections portals are highly encouraged, such as the Symbiota Collections of Arthropods Network (SCAN, http://scan-bugs.org/portal/) and the Mycology Collections data Portal for fungi (MyCoPortal, http://mycoportal.org/portal/).

Citizen science projects can mobilize thousands of participants and thus are an asset for the detection of attractive and easily recognizable species. The Lost Ladybug Project is documenting ranges, habitats, and range/habitat shifts for the North American Coccinellidae fauna through submitted photographs, which are identified by experts and entered into a database (http://www.lostladybug.org/). As such, the Lost Ladybug Project represents a major, openly available reference for coccinellid occurrences. Similarly, an online survey (http://www.ladybird-survey.org/) was launched to monitor the spread of *H*. *axyridis* in the UK while promoting the continued recording of other ladybird species. Tens of thousands of people have contributed with observations of ladybirds [59,60], providing an invaluable large-scale and long-term dataset that has been used to explore the invasion process and trends in the distribution of other ladybirds [5,6,61]. For example, using the records collated through the UK Ladybird Survey, declines in the distribution of 7 native ladybird species (of 8 assessed) have been correlated with the arrival of *H*. *axyridis* [62].

Also, *H*. *axyridis*-associated natural enemies can be monitored through citizen science programs with a local or even global perspective. An initiative in the UK to report ladybird parasitoids in 2010 (http://www.bbc.co.uk/breathingplaces/ladybird-parasites/) attracted only few contributors who, however, provided high-quality data [63]. Photographs from citizen scientists can be screened for ectoparasitic associates, such as *Hesperomyces virescens* (Fungi, Laboulbeniales) [51,64]. In this way, ladybird observations from iNaturalist and Flickr resulted in new records of the *Hesperomyces virescens* on *H*. *axyridis*, expanding the known distribution in both northern and southern directions [51]. Moving forward from these online available data, we created a website combining all available reports of the *H*. *axyridis*–*H*. *virescens* association—citizen science observations from Bugguide.com and iNaturalist, data from digital photos uploaded to Flickr, and records from the literature (http://www.beetlehangers.org/). The website currently focuses on North America, but we aim to expand both in terms of data sources (e.g., natural history collection studies) and geography.

### *Harmonia axyridis* in the Americas

*Harmonia axyridis* has an almost continuous distribution from North to South America [12]. In North America it is known from Canada [65], throughout the contiguous states of the USA [66], and Mexico [11]. In South America, *H*. *axyridis* has been reported in Argentina [29,30], Brazil [22,31], Chile [30], Colombia [21,38,39], Paraguay [44], Peru [45], Ecuador [40], Uruguay [46,47], and Venezuela [48]. According to Camacho-Cervantes and colleagues [12], the only areas where *H*. *axyridis* has not yet been found include all countries in Central America from Guatemala to Panama; and Bolivia, Guyana, and Suriname in South America. We add French Guiana to this list; to our knowledge no reports were previously known from this country. There is, however, a report of *H*. *axyridis* from Panama [43] that has gone unnoticed by the larger entomological community.

With this study, integrating reports from our own observations, museum insect collections, and online available data, we add the first reports of *H*. *axyridis* in Central America (Costa Rica, Guatemala, and Honduras), and the Caribbean (Puerto Rico). We also show a notable expansion of the known distribution ranges of *H*. *axyridis* in South America, and the consistent presence of this alien species in invaded areas. Because of technical developments in recent years, it is not surprising that 80% of all online records were made during the last 5 years. For most provinces the online records are in temporal proximity of the first published reports, although in certain provinces the online observations precede the earliest record published in the literature. In Table 1, we listed 15 countries in Central and South America for which *H*. *axyridis* has been reported in 97 provinces. Our 1,066 online observations from iNaturalist and Flickr added first records of *H*. *axyridis* for 40 provinces in 12 countries for which no records were known.

### *Harmonia axyridis* in Central America

In most reviews of global *H*. *axyridis* distribution patterns [10,12,39], Central America is either not mentioned or added as a footnote only. Camacho-Cervantes and colleagues [12] explicitly state that *H*. *axyridis* has not been reported in Central America from Guatemala to Panama (but see [43]). In Mexico, which borders Guatemala to the north, during the earliest releases of *H*. *axyridis* in 1999–2002, over 18,000,000 individuals were released in citrus plantations in Campeche, Quintana Roo, and Yucatán [67,68,69]. Interestingly, we did not find any online records from the Yucatán Peninsula but *H*. *axyridis* appears to be widely distributed in the rest of the country. *Harmonia axyridis* can move around 160–200 km/year, whereas human movement greatly accelerates the spread [10], causing a clumped distribution of records in and around municipalities.

Contrasting to the invasion in Mexico, which started in the early 2000s, the first record for Costa Rica, and one of the oldest for Latin America, is from 1988 (a specimen from San José, deposited at UCR). Several other records from the 1990s were collected in Alajuela, Heredia, and San José (Table 1), all in the highly populated Central Valley. Only one comparable old record exists: from 1989, in Chachagüí, Colombia [21]. The first releases of *H*. *axyridis* in South America occurred in 1986 in Mendoza, Argentina [70] and in 1998 in central Chile [30]. These early records from Central and Northern South America–roughly 20 years before the worldwide, large-scale spread of *H*. *axyridis*–are particularly interesting. Despite the well documented, rapid invasions in various countries with intentional releases (e.g., Argentina, Chile, Mexico, European countries) [10], the first record of *H*. *axyridis* in Colombia was from 2011 [39]. Later, based on the study of insect collections, earlier records were found dating back to 1989 [21]. Also in Costa Rica, *H*. *axyridis* was collected very early, however to our knowledge no studies were conducted monitoring its distribution in the country or its impact on ladybird community structure. The slow establishment process in both Colombia and Costa Rica indicates no intentionally releases, but more likely accidental introductions. Nevertheless, *H*. *axyridis* is very common in Colombia today [21].

Building on previous work [13], Lombaert and colleagues investigated the population structure and possible scenarios of global invasion using statistical analyses of population genetics data [71]. Most regions show similar genetic clustering (e.g., eastern North America, western Europe) but samples from South America (Brazil, Argentina, and Chile) were highly diverse in their Bayesian clustering. Individuals from Chile form a genetic unit that is dissimilar from the Argentinian and Brazilian units, both of which originated from the same introduction event. These data suggest two independent events from the eastern North American bridgehead population to South America [71]. We suggest expanding on these results and including individuals from Central America, where *H*. *axyridis* occurs already for 30 years. This is crucial if we want to understand the origins of Central American specimens.

### Distribution and “robustness” of *H*. *axyridis* in Latin America

Since the releases of *H*. *axyridis* for pest control in South America, this species is spreading continuously. By including the public in a large-scale monitoring initiative in Chile, the annual spread is logged and mapped (http://chinita-arlequin.uchile.cl/ [72]). This is reflected in our results; for all provinces in which we found records through iNaturalist and Flickr, reports were already published by Grez and colleagues partly through their monitoring program [15,30]. Other monitoring programs in Latin America are inexistent, although a recent initiative is undertaken to study the expansion of *H*. *axyridis* in Patagonia, Argentina [73]. Therefore, information regarding the distribution of this invasive species is largely limited to records in the literature. Often, only “first country records” are published [33,40], missing out on subsequent information. The records gathered in this study reflect and corroborate the currently published distribution of *H*. *axyridis* (Fig 2) and confirm the continuous presence in Latin American countries for 30 years (Costa Rica). Further, the discovery of larval and pupal stages of *H*. *axyridis* in the wild indicates established populations in Costa Rica and Honduras in Central America as well as in Argentina, Brazil, Chile, Colombia, Ecuador, Peru, and Uruguay in South America. Except for a few countries in which *H*. *axyridis* quickly became an annoyance, the large-scale invasion took place unnoticed in most of Latin America. Therefore, details on occurrences and routes of invasion require further investigations. The very old records from Colombia [21] and Costa Rica (this study) might indicate earlier events of introduction compared to those available in the literature [71].

Further, the now numerous new reports from the Neotropics (Central and South America) and the Caribbean, along with records from very hot climates [24] and high altitudes in the Andes [74], suggest a broad adaptability of *H*. *axyridis* to extreme climatic conditions. Only 10 years ago, when *H*. *axyridis* was known from 5 locations in South America (Brazil and Argentina), Poutsma and colleagues [75] modeled an index of climatic suitability based on climatic conditions of its native range. The current distribution of *H*. *axyridis* reflects astonishingly well the predicted occurrence in Central and South America. Taking into account records from Puerto Rico (this study) or Saudi Arabia [24], areas supposedly not suitable for *H*. *axyridis*, the invasive potential of this beetle becomes visible. At the same time, we note that high summer temperatures [19,20] and scarcity of prey [25,26] have been suggested to limit successful invasion of *H*. *axyridis* in Saudi Arabia. It is critical to track the worldwide invasion of *H*. *axyridis* and effects of this species on native fauna with further studies focusing on distribution and species interactions. Effective prevention mechanisms for invasive alien species are required to prevent global distribution, which goes hand in hand with effects on local or other pest fauna (e.g., *Anoplolepis gracilipes* crazy ants [76], *Hemidactylus frenatus* house geckos [77], *Blattella germanica* cockroaches [78], *Rattus norvegicus* [79]), and to create Integrated Pest Management programs for newly emerging invaders.

## Supporting information

S1 Dataset Overview of all reported observations of *H. axyridis*. All *H*. *axyridis* records from Latin America gathered during this study, with ID number, developmental stage, morph, link where applicable, geographic coordinates, collecting date, locality (country & province/department), and source (iNaturalist, Flickr, museum collection, research paper).
(XLSX)
